# The "Diphtheria House" at Zurich

**Published:** 1890-03-01

**Authors:** 


### The "Diphtheria House" at Zurich.

Across the court-yard, behind the body of the Hospital Buildings, stands an unpretentious stone erection—unpretentious at least from this side. It is the "Diphtheria House," where all cases of diphtheria admitted are at once placed in isolation. A handsome vestibule leads forward through a glass door into what might be a conservatory for exotics in some princely mansion. The floor is of particoloured pebbles arranged in patterns in a superior asphalt, and is firm, cleanly, and elegant, but rather too slippery for convenience. What has been termed the conservatory is an apartment walled and roofed in entirely with glass set in a framework of thin iron ribs. The size is about that of a dining-room in large mansions, only more lofty, and constructed on a square base. Being literally a glass house there is ample illumination. The wall throughout is double, with an inner and outer casement, so that when one of the inner panes is opened the cool air circulating between the two walls is admitted to an interchange with the impure air of the apartment. During the summer season both inner and outer windows would be opened. One finds these double windows, be it remembered, in ordinary dwelling-houses in Zurich, for the winter is very cold in these elevated regions of the Continent. There was space for six beds, only one of which was occupied by a lad of three years. The bed was of metal, supported on thin brass posters, with a chainwork to support the mattress and bed-clothes. Near was a small spray apparatus which distributed steam—that is to say, pure water. The sufferer was very ill and prostrate, his face bedewed with drops of water from condensation of the steam. A little further from him towards the door was a large steam boiler intended to distribute the steam—pure, not medicated—to the whole room. Experience has shown that the value of these apparatus is, at all events in the opinion of the House Surgeon, dubious: for, paradoxical as it may seem, the atmosphere of the apartment is rendered drier and hotter than ordinary. At the same time, it may be remarked that no use was made in treatment of local applications, such as lactic acid, to the throat and pharynx; though, so long as gargling was practicable lotions containing 2 per cent. dilute hydrochloric acid, were prescribed.

Delighted with the airiness, lighting, and perhaps the novelty of this ward, one retired and found in the wings to the right and left accommodation for convalescent patients. In one ward of seven beds, furnished as just described, but with ordinary painted walls, lay two tracheotomy cases now convalescent and in good spirits as they played with dolls and Christmas nic-nacs, and smiled at the kindly notice of Professor Kroenlein, the Surgeon-in-Charge. In a similar ward in the other wing across the entrance hall, lay two other patients who seemed almost able to return to their homes.

There were corresponding wards upstairs. An operating theatre, on the ground floor, was noteworthy for a new and powerful lamp, with a large silvered reflector two feet across, suspended immediately over the table. With its assistance operators would not shrink from a tracheotomy at any hour of the night. A shaft leading down to the cellars into which all soiled dressings and infected materials were at once thrown, stood conveniently near the table. There was a bath-room fitted with all the most modern appliances. Finally, there were rooms for investigation and research, together with apartments on the floor above for keeping the various specimens, which were gradually forming a museum of all that was pathologically most interesting in relation to diphtheria.

